# Ultrafast Metrology through Nonlinear Plasmonic Metasurfaces

**DOI:** 10.1021/acs.nanolett.6c01318

**Published:** 2026-06-06

**Authors:** Binod Bhatt, M. Akeel Faris, Chunlei Guo

**Affiliations:** The Institute of Optics, 6927 University of Rochester, Rochester, New York 14627, United States

**Keywords:** metasurfaces, ultrafast optics, ultrafast metrology, nonlinear optics, pulse characterization, autocorrelators, frequency-resolved optical gating

## Abstract

Conventional ultrafast pulse characterization relies on bulk nonlinear crystals. While effective, these crystals are constrained by strict phase-matching conditions that restrict bandwidth, and their bulky sizes hinder on-chip integration. Here, we present a plasmonic metasurface platform that addresses these limitations by enabling second-harmonic generation over a subwavelength propagation. Our plasmonic nanoantennas with broken structural symmetry are designed to support a broadband electric-dipole resonance centered around 830 nm. For the first time, we introduce metasurfaces to ultrafast metrology by demonstrating both a metasurface-based interferometric autocorrelator for pulse intensity characterization and interferometric frequency-resolved optical gating for complete intensity-phase characterization. Furthermore, we characterize the device’s photothermal resistance under extended pulsed excitation, establishing the operational fluence level for continuous metrology operation. This work paves the way for the development of broadband and chip-scale metrology systems.

## Graphical abstract

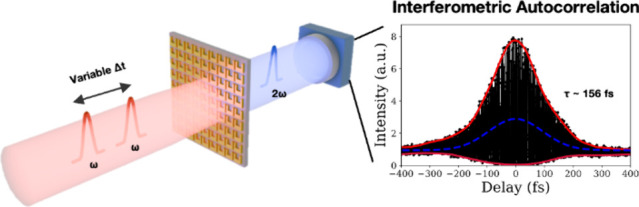

The miniaturization of optical systems has become increasingly important as modern photonics moves toward compact, portable, and low-power platforms capable of performing advanced functions outside traditional laboratory environments. This need is largely unmet in ultrafast optics, where subpicosecond and femtosecond laser pulses^1^ are central to applications including optical communications,^2,3^ ultrafast spectroscopy,^4,5^ multiphoton imaging,^6,7^ and high-field physics.^8,9^ Furthermore, ultrafast pulses are essential for advanced laser processing,^10,11^ enabling the functionalization of material surfaces and the creation of engineered optical, structural, and wetting properties.^10,12–14^ Reliable use of these techniques requires accurate temporal and spectral pulse characterization, commonly achieved through autocorrelators or full intensity-phase characterization tools, such as Frequency-Resolved Optical Gating (FROG) and Spectral Phase Interferometry for Direct Electric-field Reconstruction (SPIDER).^15–18^

At the core of these measurement techniques are nonlinear optical materials, such as Beta Barium Borate (BBO) and other traditional bulk crystals.^19^ While effective, these media impose fundamental constraints that hinder miniaturization: they require strict angular phase-matching that restricts spectral bandwidth, introduce material dispersion capable of distorting broadband pulses, and possess macroscopic dimensions incompatible with chip-scale integration. These limitations create a critical bottleneck for developing compact ultrafast metrology systems. To mitigate this, alternative nonlinear platforms have been implemented in ultrafast metrology. Nonlinear photonic crystals^20,21^ were utilized specifically to relax the strict angular phase-matching constraints of nonlinear crystals, yet they remain bulky for true on-chip integration. In parallel, unstructured metal films^22,23^ were explored to enable compact and broader band diagnostics, but they still suffer from inherently weaker efficiency due to symmetry restrictions.^19,24^

Nonlinear plasmonic metasurfaces offer a superior alternative to overcoming these limitations. By utilizing localized surface plasmon resonances (LSPRs) to strongly enhance the local electric field, they enable efficient second-harmonic generation (SHG).^25–28^ Furthermore, their deeply subwavelength thickness effectively relaxes strict phase-matching requirements^29^ (see Supporting Information, Figure S1) and ensures inherent compatibility with planar photonic integration, establishing them as a compelling platform for miniaturized ultrafast diagnostics. Figure 1 illustrates the proposed metasurface-based autocorrelation technique, where varying the delay between a collinear pulse pair yields an SH signal that allows extraction of the fundamental pulse duration.

**Figure 1:**
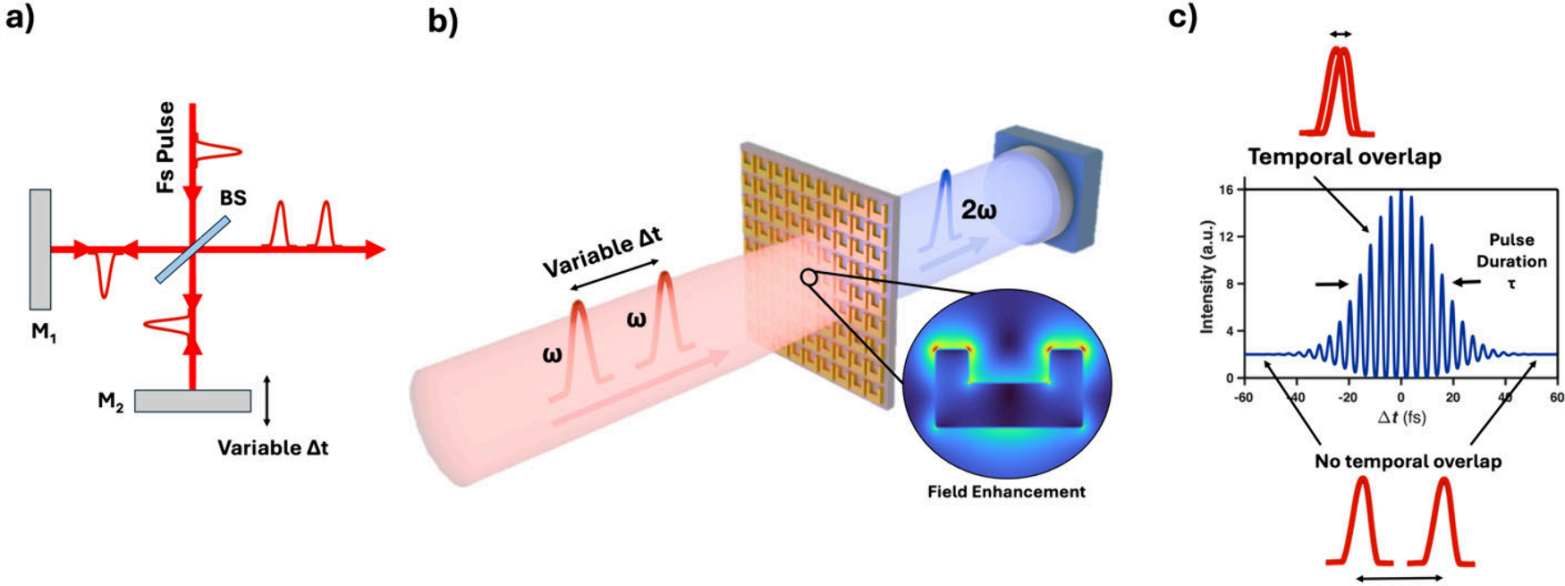
Schematic illustration of the metasurface-based autocorrelation technique. (a) A Michelson interferometer, comprising reflecting mirrors (*M*_1_, *M*_2_) and a beam splitter (BS), generates a delayed collinear pulse (ω) pair. (b) This pulse pair excites localized surface plasmon resonances in a nonlinear metasurface. The resulting electric field enhancement (inset) drives efficient second-harmonic (2ω) generation. Panel (c) illustrates that by recording the SH intensity as a function of temporal overlap an autocorrelation trace is produced to extract the ultrafast pulse duration (τ).

In this work, we demonstrate a metasurface composed of gold (Au) nanoantennas with broken structural symmetry, optimized via nonlinear scattering theory^30^ for robust SHG. We integrated the metasurface into an interferometric setup to perform both fringe-resolved autocorrelation and Interferometric FROG (IFROG) trace acquisition. By applying Fourier filtering and the Principal Component Generalized Projections (PCGP) algorithm, we successfully reconstructed the full complex electric field. The autocorrelation trace exhibited the expected 1:8 peak-to-background contrast, while the IFROG measurement retrieved a 156 fs pulse with a distinct quadratic phase profile. These results, validated against a commercial Grenouille, confirm the device’s accuracy for pulse characterization. Furthermore, we demonstrated the device is photothermally stable at the fluences needed for pulse characterization, demonstrating its durability for practical, chip-scale ultrafast metrology.

While inversion symmetry can be broken extrinsically through oblique incidence, our metasurface design is based on asymmetric nanoantennas, to enable efficient second-order nonlinear generation otherwise forbidden in centrosymmetric media.^31,32^ Furthermore, this asymmetry provides access to polarization-selective, higher-order localized surface-plasmon modes with strong, geometry-confined field enhancement that a perfectly symmetric structure cannot support. While nonlinear metasurfaces using similar split-ring resonator geometries have been explored across visible, near-infrared, and telecommunication wavelengths, most of the demonstrations rely on magnetic-dipole dominated resonances, particularly in the 1.0–1.6 μm wavelength range.^33,34^ In contrast, the U-shaped metasurface presented here utilizes the electric-dipole (ED) resonance present in the 830 nm spectral window, well-separated from the structure’s fundamental magnetic resonance.

For excitation with polarization along the principal symmetry axis (y-pol) under normal incidence, the metasurface supports a dominant electric-dipole (ED) resonance as shown by the field distribution (*E*_*z*_) normal to the metasurface plane in Figure 2a­(i). It is characterized by an antisymmetric charge distribution with opposite polarities at the ends of the two vertical arms (Figure 2a­(ii)). This charge separation along the two arms behaves as a short dipole, where the field enhancement is strongest at the arm tips or outer corners, and decreases toward the central region as shown in Figure 2a­(iii). The peak electric field enhancement for y-pol excitation is ∼13 at the edge of the geometry. In contrast, for excitation with polarization perpendicular to the principal symmetry axis (x-pol), a higher-order electric-quadrupole (EQ) resonance is excited (Figure 2a­(iv)), exhibiting a four-lobe charge pattern (Figure 2a­(v)). This mode results in the opposite ends of the nanoantenna arms being oppositely charged and concentrates the electric field at the corners of the arms, producing a stronger enhancement of ∼18 (Figure 2a­(vi)). While the narrowband EQ dark mode provides intense near-field confinement ideal for Fano-resonance-based application such as sensing, it plays a negligible role in far-field emission for our measurements. Therefore, SHG from our metasurface predominately comes from the bright ED mode, and we next examine the geometric control of its broadband spectrum.

**Figure 2:**
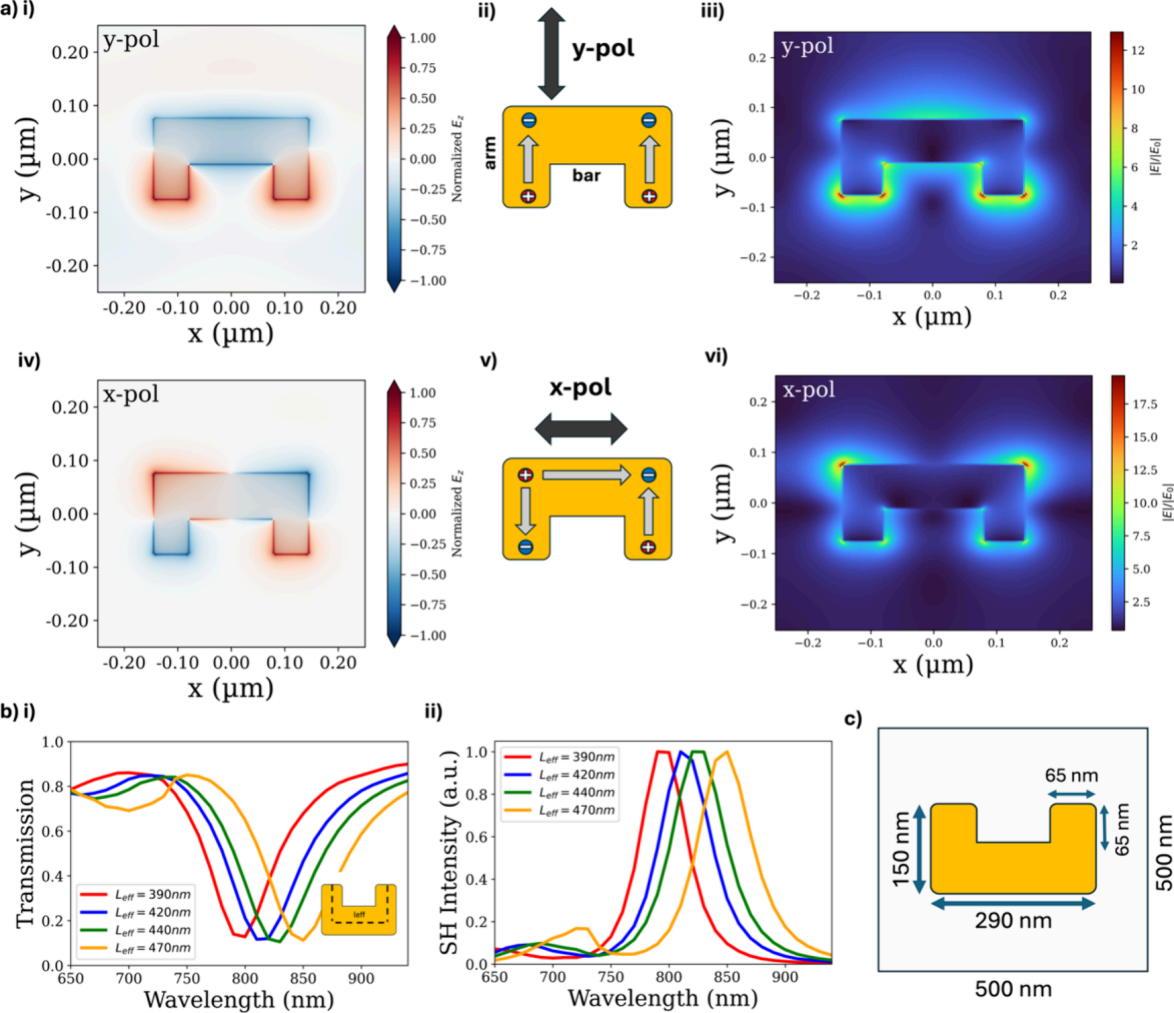
Polarization-dependent resonances and spectral tuning in the U-shaped asymmetric metasurface. (a) Mode profiles at normal incidence near the 830 nm wavelength. (i) Simulated *E*_*z*_ map showing an electric-dipole (ED) resonance for y-polarized incident light. (ii, v) Schematics of the metasurface geometry and charge distribution for ED and EQ, respectively. (iii, vi) Near-field electric field enhancement for ED and EQ modes calculated at the surface of the structure, respectively. (iv) Simulated *E*_*z*_ map showing an electric-quadrupole (EQ) resonance for x-polarized incident light. (b) Spectral response versus metasurface effective length *L*_eff_. (i) Transmission spectra for increasing *L*_eff_ (390–470 nm). (ii) Normalized SHG spectra that tracks the transmission dip as *L*_eff_ varies. (c) Optimized unit-cell geometry used in simulations: vertical and horizontal nanorod lengths of 150 and 290 nm, respectively, period 500 nm, and 35 nm thickness.

In Figure 2b­(i) we have plotted transmission vs wavelength for different metasurface effective lengths *L*_eff_. The observed transmission dip is a direct consequence of the LSPR modes that effectively couple with the driving electric field, leading to enhanced absorption and scattering of the incident light. The spectral position of this resonance is tunable by varying *L*_eff_. Consistent with the linear relationship established in Figure S3b, as *L*_eff_ increases, the transmission dip redshifts while its bandwidth remains essentially unchanged. Consequently, we select *L*_eff_ = 440 nm to match the 830 nm wavelength of our mode-locked oscillator.

The nonlinear response of the U-shaped asymmetric metasurface can be modeled using nonlinear scattering theory through the microscopic linear response of the metasurface.^30^ This theory assumes a local nonlinear susceptibility tensor at the metal-air interface and uses the Lorentz reciprocity theorem to quantify the nonlinear emission (see Supplementary Note 2). The second-harmonic emission *E*_*SH*_(2*w*) is described as

$$E_{SH}^{2\omega }\propto \iint \left(\chi_{nnn}E_{n}^{\omega }E_{n}^{\omega }E_{n}^{2\omega }+\chi_{ntt}E_{t}^{\omega }E_{t}^{\omega }E_{n}^{2\omega }+\chi_{ttn}E_{n}^{\omega }E_{t}^{\omega }E_{t}^{2\omega }\right)d^{2}r$$

where *n* and *t* are the normal and tangential unit vectors along the surface of the metal, respectively. *χ*_*nnn*_, *χ*_*ntt*_, and *χ*_*ttn*_ denote the local nonlinear susceptibility component where the first subscript represents the component of the linear electric field for the second harmonic frequency and the latter two subscripts represent the components for the fundamental frequency. The equation evaluates the overlap integral between the microscopic nonlinear polarization, which is determined by the linear fields at the fundamental and the second harmonic modes, to compute the far-field nonlinear emission.

As suggested by the governing equation for SHG above, the nonlinear response is directly proportional to the square of the fundamental electric field. LSPR modes are known to provide significant local enhancement of this fundamental field, which is expected to strongly boost the nonlinear response. Figure 2b­(ii) clearly verifies this principle by illustrating the direct correlation between the linear and nonlinear responses. The simulated normalized SH intensity, plotted for the *L*_eff_ values from Figure 2b­(i), displays a distinct peak at the fundamental resonance and precisely tracks the linear transmission dip as *L*_eff_ is varied.

We perform 3D finite-difference time-domain (FDTD) simulations using Tidy3D to design and optimize the metasurface^35^ (see Supplementary Note 3 for simulation details). The optimized geometry uses vertical and horizontal nanoantenna lengths of 150 and 290 nm respectively, with a thickness of 35 nm (Figure 2c).

The metasurface was fabricated through a metal lift-off process and electron beam lithography at the Integrated Nanosystems Center (University of Rochester) (Supplementary Note 4). Figure 3a shows scanning electron microscopy (SEM) image of the fabricated array. To account for geometric deviations observed during fabrication, the initial model was refined by incorporating a 5 nm radius of curvature at the vertices. This adjustment compensates for corner rounding effects, ensuring the simulated model more accurately reflects the physical experimental geometry.

**Figure 3:**
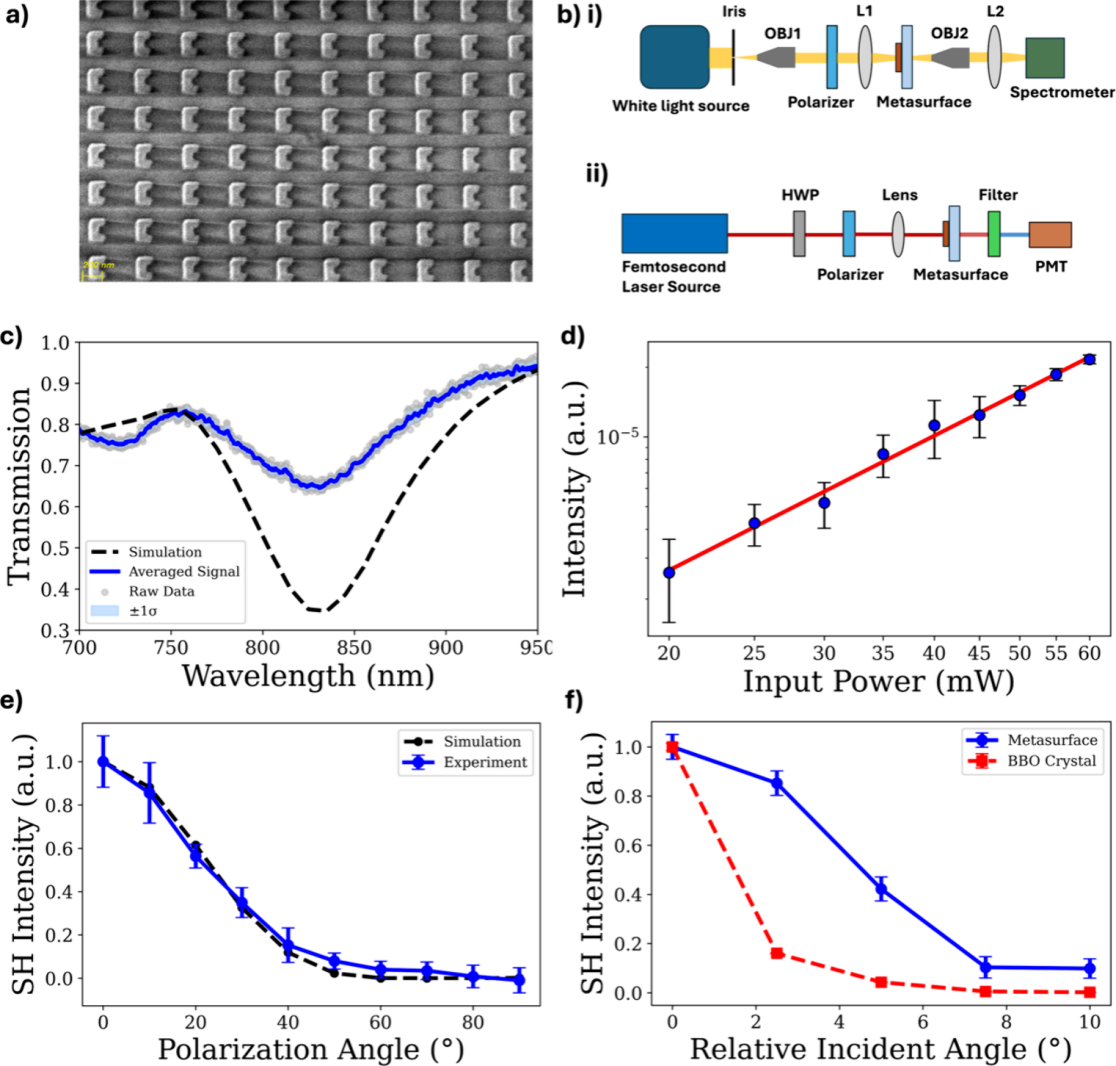
Fabrication, measurement setups, and results. (a) SEM image of the fabricated metasurface. (b) (i) Schematics of the experimental setup for measuring the transmittance of the metasurface. OBJ: objective lens, P: polarizer, L: lens. (ii) Schematic of the experimental setup for measuring the second harmonic signal of the metasurface. HWP: half wave plate, PMT: photomultiplier tube. (c) Measured (solid) and simulated (dashed) transmission spectra showing close agreement between the two. (d) Second-harmonic intensity versus incident pump power. The solid line is a quadratic fit to the SHG signal. (e) Second-harmonic intensity at different linear polarization angle. (f) Normalized second-harmonic intensity versus incident angle for the metasurface and a bulk BBO crystal. Each trace is independently normalized to its own peak to compare angular tolerance, as the BBO crystal and our metasurface have different signal strengths.

For linear characterization (Figure 3b­(i)), we measured transmission under y-polarized light. White light from a quartz–tungsten–halogen lamp (Thorlabs SLS301, 360–2700 nm) was collimated, polarized (Melles Griot 03 PTH 112/C, 350–2300 nm), and focused onto the sample with a plano-convex lens (L1). Transmitted light was collected by an objective (OBJ2), relayed by lens (L2), and sent to a fiber-coupled spectrometer (Photon Control SPM001, 350–1000 nm). The obtained spectra were normalized to the bare quartz substrate.

For nonlinear characterization (Figure 3b­(ii)), we used a Ti:sapphire oscillator (KM Laboratories Griffin, 830 nm, ∼93 MHz). The beam passed a half-wave plate and polarizer to set y-polarization, then was focused onto the metasurface. Second-harmonic light copropagating with the fundamental was isolated by a narrowband filter at 2ω, detected by a photomultiplier tube (PMT), and read out with a lock-in amplifier modulated by a chopper.

Figure 3c presents a direct comparison between the measured and simulated transmission spectrum. A concordance is evident, as the experimental data accurately reproduces the spectral position and overall bandwidth of the resonance dip predicted by the simulation. The measured dip is slightly shallower than the simulated one, a discrepancy which we attribute to fabrication imperfections not captured in the ideal numerical model. This excellent overall agreement confirms the validity of our design and the successful fabrication of the structure.

For the nonlinear signal characterization, we first verified its origin with a power-dependence measurement. Figure 3d plots the signal strength as a function of the average incident laser power. The data fits with a quadratic function, showing excellent agreement and demonstrating the expected behavior for a SHG process. This quadratic dependence is strong evidence of the signal’s SHG origin from the metasurface.

Next, to validate our nonlinear simulation, we characterized the SHG response as a function of the incident polarization angle at a fixed 830 nm wavelength (Figure 3e). Here, 0° corresponds to the y-polarization that excites the electric dipole mode, while 90° corresponds to the orthogonal, nonresonant x-polarization. As predicted, the measured intensity is maximized at 0° and minimized at 90°. The experimental data shows an excellent match with the simulation, confirming the predictive accuracy of our simulation.

To quantify the angular robustness of our platform, we compared the SHG response of the metasurface against a typical BBO crystal (Figure 3f). Because nonlinear conversion efficiency scales fundamentally with the interaction length, the macroscopic thickness of the BBO crystal naturally yields a much higher SH signal than that of our subwavelength-thick metasurface. Here, we study the relative signal change as a function of the incident angle. As shown in Figure 3f, where each signal is individually normalized to its respective peak to facilitate this direct comparison, the BBO signal drops by more than 80% with a detuning of just 2.5° from the optimal angle due to its strict phase-matching requirements. In contrast, the metasurface response remains stable, experiencing less than a 20% reduction under identical conditions, confirming its immunity to strict angular alignment constraints. We note that the angular dependence of the BBO crystal depends on its phase matching condition that arises from momentum conservation, while the angular dependence of our metasurface arises from an implicit plasmonic resonance matching condition. We can see from Figure 3f that this resonance matching constraint is more relaxed than the momentum conservation required by birefringent crystals.

To demonstrate the practical usage of our metasurface, we use it as the nonlinear medium to perform a fringe-resolved interferometric autocorrelation (IAC) of our Ti:sapphire oscillator (KM Laboratories Griffin, 830 nm, ∼93 MHz). Detailed descriptions of the experimental Michelson-type interferometer setup are provided in Supplementary Note 5. The metasurface-based IAC is used to characterize the pulse duration of the oscillator. The resulting trace in Figure 4a displays the theoretical 1:8 peak-to-background ratio (see Supplementary Note 6), indicative of stable, fringe-resolved autocorrelation. A Gaussian fit to the trace envelope yields a pulse duration of ∼156 fs.

**Figure 4:**
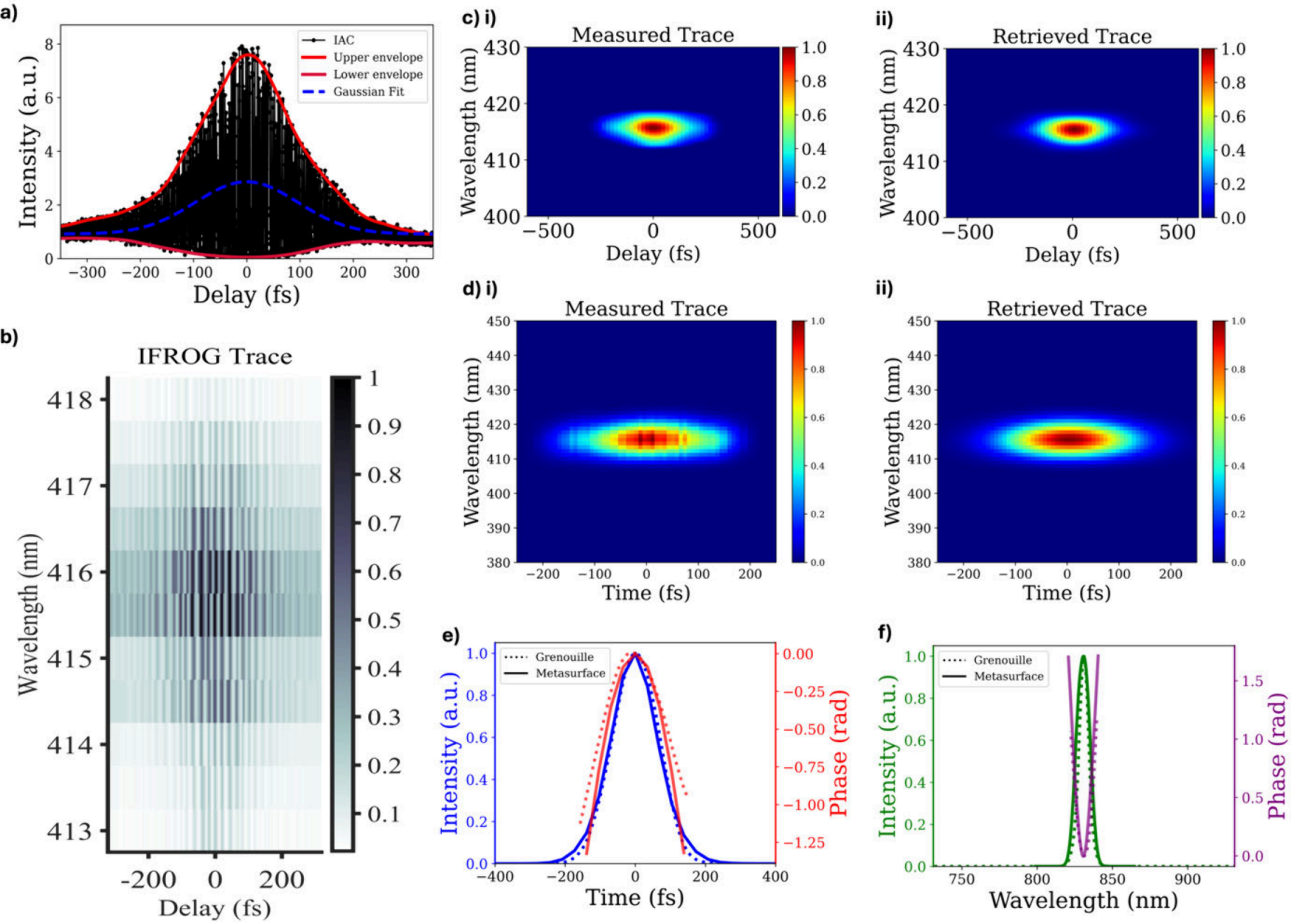
Complete pulse characterization. (a) Measured fringe-resolved interferometric autocorrelation (IAC) trace showing the expected 1:8 peak-to-background ratio; a Gaussian-envelope fit gives ∼156 fs. (b) Raw measured interferometric FROG (IFROG) trace. (c) (i) Background-free SHG-FROG spectrogram extracted via Fourier-domain filtering of the raw trace. (ii) Retrieved spectrogram using the PCGP algorithm; the reconstruction error is 1 × 10^–5^. (d) Independent commercial characterization using a Grenouille 8–9 USB: (i) measured and (ii) retrieved traces. (e) Reconstructed temporal intensity and phase profiles comparing the metasurface (∼156 fs) and Grenouille (∼157 fs); both show a distinct quadratic phase indicative of chirp. (f) Retrieved spectral intensity and phase profile comparison of the metasurface and Grenouille.

While IAC characterizes the pulse duration, it does not provide the full complex electric field. To obtain complete temporal and spectral information, we implemented Interferometric Frequency-Resolved Optical Gating (IFROG) using the nonlinear metasurface. Figure 4b displays the raw experimental IFROG trace obtained using the nonlinear metasurface. To extract the standard background-free SHG-FROG spectrogram, we applied Fourier-domain spectral filtering along the delay axis.^36^ The central DC band was isolated using a super-Gaussian filter to eliminate interferometric fringes, and the constant background was subtracted to yield the final SHG-FROG trace in Figure 4c­(i). This spectrogram was processed using the Principal Component Generalized Projections (PCGP) retrieval algorithm,^37^ implemented in the open-source Python library.^38^ A detailed description of the algorithm, including synthetic validation benchmarks and the error convergence profile, is provided in the Supplementary Note 7. The algorithm converged to a low FROG error of 1 × 10^–5^ and the retrieved trace (Figure 4c­(ii)) shows close agreement with the measured data.

To validate these metasurface-based results, we performed independent measurements using a commercial Grenouille (Swamp Optics Grenouille 8–9 USB). The corresponding measured and retrieved SHG-FROG traces from the Grenouille are shown in Figure 4d­(i) and (ii), respectively, supporting the accuracy of our metasurface-based technique. The reconstructed temporal intensity and phase profiles are presented in Figure 4e, where the retrieved profiles are overlaid with the independent Grenouille measurements. The retrieval yields a pulse duration (fwhm) of 156 fs. Notably, the retrieved temporal phase exhibits a distinct quadratic profile, indicating the presence of chirp, consistent with Grenouille measurements. Finally, the spectral domain characterization is shown in Figure 4f, again showing a close agreement with the Grenouille data. The retrieved spectral bandwidth is 10 nm, showing good agreement with the 8 nm bandwidth measured independently using a fiber spectrometer. Together, the spectral consistency, low retrieval error, and stable convergence confirm accurate pulse reconstruction using the metasurface-based IFROG.

To evaluate the device’s photothermal stability, we fabricated a batch of control arrays using the established recipe (Supplementary Note 4). We monitored both the optical performance and the structural integrity of the device during an extended period of pulsed excitation. Figure 5a presents the SH intensity generated by the metasurface, measured over 1- and 2-h continuous exposures at a peak operating fluence of ∼0.22 mJ/cm^2^ and a peak intensity of ∼1.40 GW/cm^2^. Each signal is normalized independently to its respective global average. The SH signal remains mostly stable throughout the measurement window, with fluctuations confined to within ±5%. This stability is further verified by the linear transmission spectra (Figure 5b), which exhibits no significant resonance shift or spectral broadening that would typically indicate thermal melting or structural reshaping. We note a more pronounced resonance dip for this sample due to batch-to-batch variation during fabrication.

**Figure 5:**
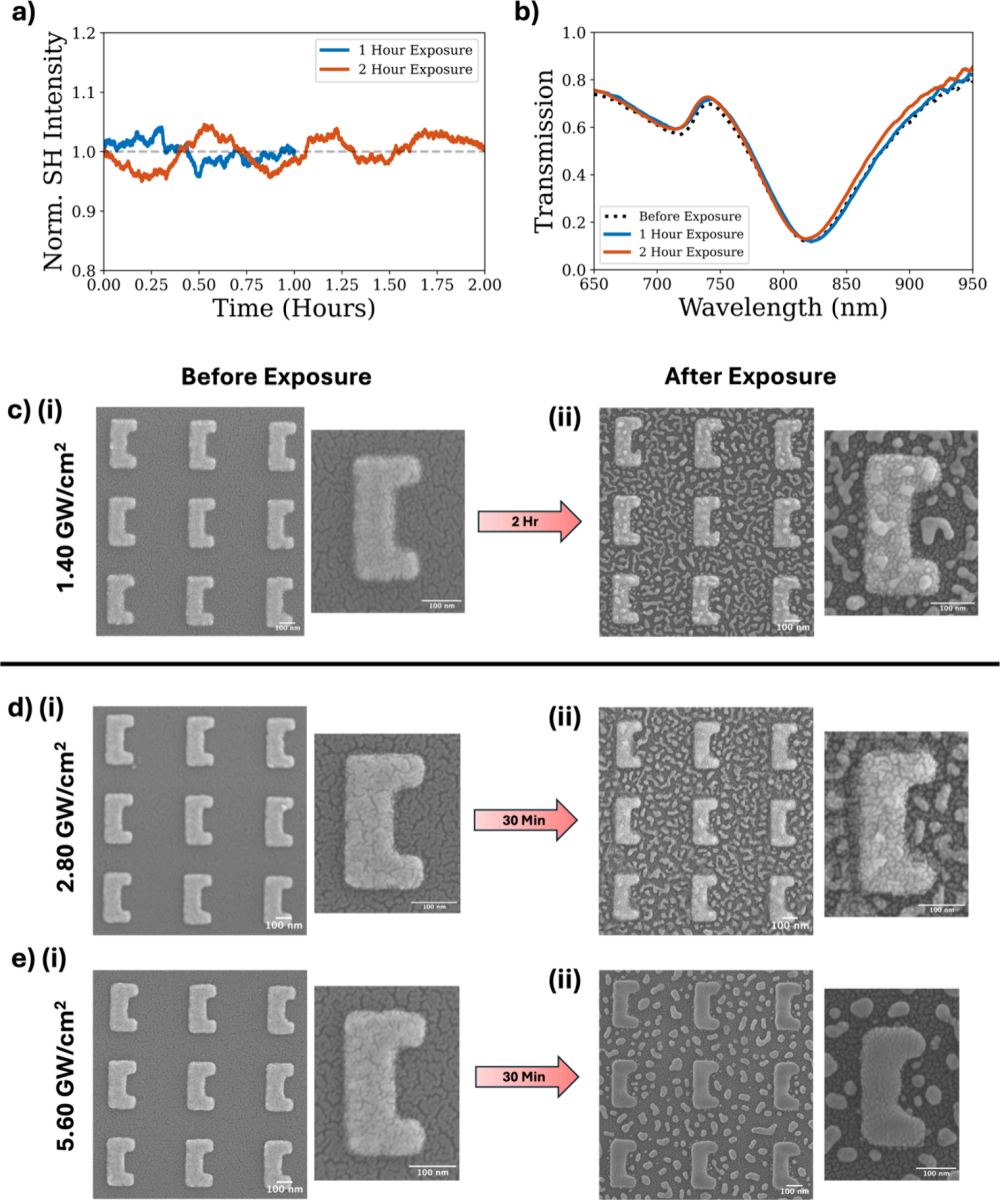
Photothermal stability and damage threshold analyses. (a) SH intensity generated by the metasurface, measured over 1- and 2-h continuous exposures at an operating peak fluence of ∼0.22 mJ/cm^2^ (corresponding to a peak intensity of ∼1.40 GW/cm^2^). Each data set is normalized independently to its respective global average. (b) Plasmonic transmission spectra before and after exposure under these conditions. (c) SEM images of a control array before (i) and after (ii) the 2-h exposure; the structures remain intact. (d, e) SEM imaging of the higher fluence 30 min exposures. The metasurface largely sustains its geometry at the peak fluence of ∼0.44 mJ/cm^2^ (∼2.8 GW/cm^2^) but undergoes significant photothermal melting at ∼0.88 mJ/cm^2^ (∼5.6 GW/cm^2^).

To confirm the absence of structural deformation, SEM was performed. Our metasurface consists of Au resonators on a quartz substrate. The quartz substrate accumulates charges during SEM imaging which significantly affects resolution. To mitigate this charge accumulation, a 5 nm Au layer was deposited on the sample prior to laser exposure to visualize the initial shape of the nano resonators. The resulting image is shown in Figure 5c­(i), with the inset detailing the geometry of an individual resonator. Following a 2-h exposure, the sample was again coated with a 5 nm Au layer to dissipate charge and enable clear post-exposure imaging. The resulting SEM image (Figure 5c­(ii)) reveals that the nanoantennas remain structurally intact, without any notable deformation along the arms or the corners. We note that the nanometer-scale fine granular features visible on and between the structures in Figure 5c­(ii) are purely the artifacts from melting of the 5 nm Au coating used for imaging and are not indicative of thermal damage to the resonator itself. Furthermore, the peak fluence of ∼0.22 mJ/cm^2^ used in this stability measurement far exceeds the required fluence level (∼88 μJ/cm^2^) for ultrafast characterization, demonstrating a long-term device stability during standard operation.

Finally, to determine the fluence limitation of the metasurface and establish an operational threshold for ultrafast metrology, we doubled the fluence level to ∼0.44 mJ/cm^2^ (∼2.8 GW/cm^2^) for a 30 min exposure. As shown in Figure 5d, this appears to be the damage threshold corresponding to the onset of a possible structural change. When we further doubled the fluence level to ∼0.88 mJ/cm^2^ (∼5.6 GW/cm^2^) for another 30 min exposure as shown in Figure 5e, we clearly observe the photothermal melting of the metasurface structures. Through these tests, we determine that ∼0.44 mJ/cm^2^ corresponds to the damage threshold, while below that the device should operate stably long-term. For applications requiring even higher fluences, it is necessary to utilize more robust refractory plasmonic materials such as titanium nitride (TiN), zirconium carbide (ZrC), and tungsten (W).^39^ TiN nanoantennas, for example, have already been demonstrated to outperform their gold counterparts by sustaining peak intensities an order of magnitude higher without melting.^40^

In summary, we have introduced a nonlinear metasurface-based pulse characterization technique that overcomes the bandwidth and integration limitations of traditional bulk crystals. By exploiting the symmetry-broken geometry of gold nanoantennas, we accessed a broadband electric-dipole resonance that enables robust second-harmonic generation by relaxing strict phase-matching constraints. We demonstrated the platform’s high-fidelity performance by recording high-contrast interferometric autocorrelation traces and further extending the technique to full-field IFROG retrieval to completely characterize the pulse. Importantly, the device exhibits strong photothermal resistance at a fluence level much higher than what is needed for metrology measurements. This study validates the use of metasurfaces for quantitative ultrafast metrology. Looking forward, this platform paves the way for fully integrated metrology systems, where metasurfaces, waveguides, and detectors can be cointegrated on a millimeter-scale chip.

## Supplementary Material



## Data Availability

The data sets generated during and/or analyzed in this study are available from the corresponding author upon reasonable request.

## Notes

Detailed comparisons of phase-matching conditions for metasurfaces versus BBO, analytical derivations of nonlinear scattering theory, comprehensive simulation and optimization parameters, device fabrication recipe, experimental setup for ultrafast pulse characterization, mathematical derivations for the interferometric autocorrelation peak-to-background ratio, and verification of the PCGP-based retrieval algorithm (PDF)
