# Supplementary material for: Ultrafast Metrology through Nonlinear Plasmonic Metasurfaces
Source: Nano Lett. 2026 Jun 6;26(23):7665–72. doi: 10.1021/acs.nanolett.6c01318 (PMC13281516; doi:10.1021/acs.nanolett.6c01318)
Supplement: Supplementary file 1 [file nl6c01318_si_001.pdf]

Supplementary Information for

Ultrafast Metrology through Nonlinear Plasmonic Metasurfaces

*Binod Bhatt, M. Akeel Faris, and Chunlei Guo**

The Institute of Optics, University of Rochester, Rochester, New York 14627, United States

*Corresponding author email: guo@optics.rochester.edu

Supplementary Note 1: Comparison of Phase Matching in Bulk Crystals vs. Metasurfaces

For second harmonic generation (SHG) in a bulk crystal of interaction length L, the second-harmonic signal intensity grows quadratically with length but is modulated by the phase mismatch $\Delta k = k_{2\omega} - 2k_{\omega}$ [1]. The intensity scales according to the standard solution for the nonlinear wave equation:

$$I_{SHG} \propto L^2 \mathrm{sinc}^2\left(\frac{\Delta k L}{2}\right) \quad (S1)$$

As a result, thick crystals demand precise birefringent phase matching and angular tuning to ensure $\Delta k \approx 0$. Furthermore, for broadband ultrafast pulses, the finite phase-matching bandwidth of a thick crystal can lead to spectral filtering and temporal broadening (Group Velocity Mismatch). In contrast, the subwavelength array of a metasurface creates a dramatic electric field enhancement over a much shorter scale than the coherence length, $L_c = \pi/\Delta k$. **Figure S1** plots the normalized second harmonic efficiency as a function of propagation distance. In the shaded region ($L \ll L_c$), representing the regime where a metasurface operates, the sinc function is approximately unity [2]. Consequently, the phase-mismatch term remains negligible, and the process is effectively phase-matched for a broad range of wavelengths and angles. For a typical BBO crystal where $L \gg L_c$, the phase mismatch accumulates rapidly along the propagation length. Even near the optimal Type-I phase-matching angle ($\theta = 29.2°$ at 800 nm), a slight detuning of $\pm 0.15°$ or a shift in wavelength significantly reduces the conversion efficiency. The metasurface, being free from these constraints, provides a broadband, alignment-free platform ideal for characterizing ultrashort pulses.

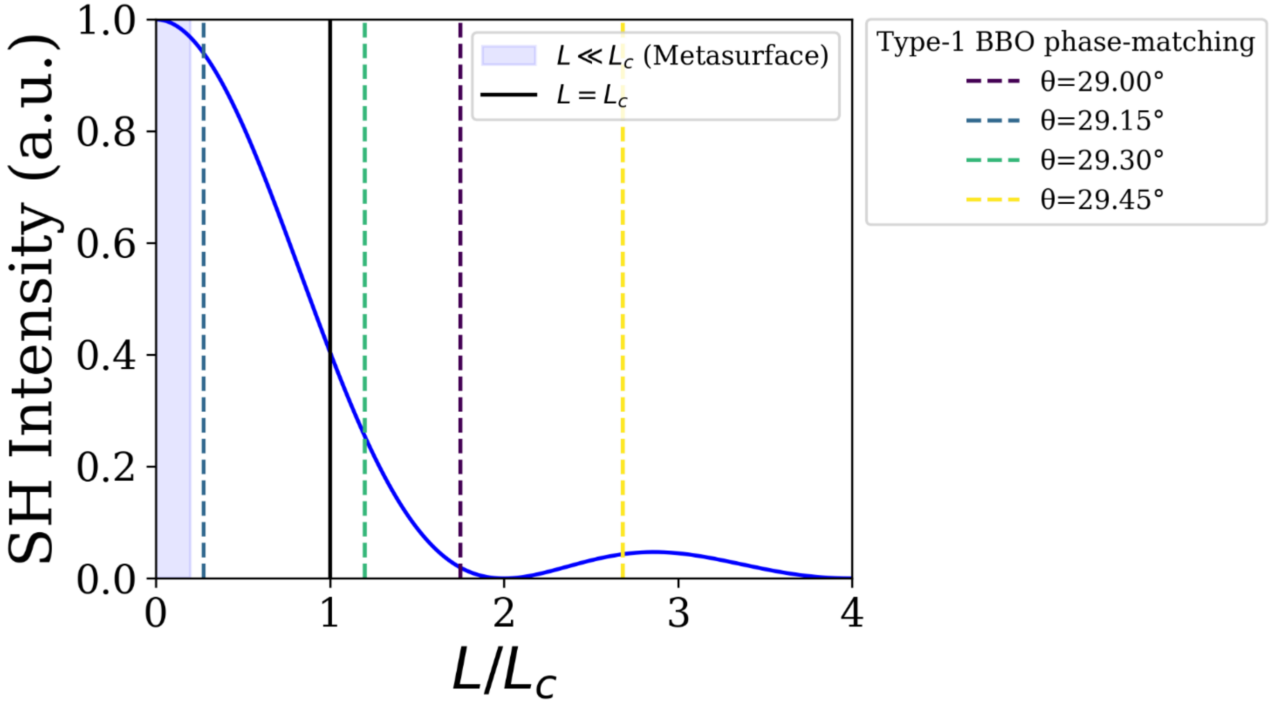

Figure S1. Dependence of SH intensity on normalized interaction length L/L_c, where $L_c = \pi/|\Delta k|$ is the coherence length.

Supplementary Note 2: Derivation of Surface Second Harmonic Emission

We utilize the Lorentz reciprocity theorem to calculate the nonlinear emission of the metasurface from its linear optical response at the fundamental and second-harmonic frequencies [3]. This framework avoids direct nonlinear simulation by relating the radiated emission to the spatial overlap between the pump-induced polarization and a reciprocal test field propagating from the detector.

Consider two electromagnetic states within a linear, reciprocal medium: a current source J_1 that generates an electric field E_1, and a second source J_2 that generates a field E_2. The Lorentz reciprocity theorem establishes a fundamental symmetry between these interactions, stating that the coupling of field E_2 with source J_1 is identical to the coupling of field $E_1$with source J_2, as shown in equation $S2$.

$$\int J_1 . E_2 dV = \int J_2 . E_1 dV \qquad (S2)$$

We begin by defining the source of the nonlinear emission. In our simulation, the fundamental electric field*s* $E^{\omega}(r)$ induced at a position coordinate r on the metal surface generates a nonlinear surface polarization. This polarization produces an equivalent surface current density $K^{2\omega}(r)$ oscillating at the second-harmonic frequency.

$$K^{2\omega}(r) = j2\omega P_{surf}^{2\omega}(r) \approx j2\omega\epsilon_0\chi_{surf}^{(2)}[E^{\omega}(r)]^2 \qquad (S3)$$

To analytically link this localized surface source to the far-field radiation detected by the observer, we invoke the theorem defined above. We introduce a hypothetical test current J_2 at the detector location, which acts as the source of our reciprocal state. This test source generates a plane

wave at the second-harmonic frequency (2ω), establishing the local electric field distribution $E^{2\omega}(r)$ across the surface of the metasurface. Since the nonlinear generation is confined to the metal-dielectric interface, the interaction volume is reduced strictly to a surface integral. Normalizing out the test source term, we find that the far-field nonlinear emission E_{SH} is proportional to the spatial overlap of the induced nonlinear polarization and the reciprocal linear field:

$$E_{SH} \propto \int_{S_{meta}} P_{surf}^{2\omega}(r) \cdot E^{2\omega}(r)\, dS \qquad (S4)$$

To evaluate the overlap integral, we decompose the electric fields into components normal ($\hat{n}$) and tangential ($\hat{t}$) to the metal surface. For an isotropic metal interface, the total polarization vector is constructed from three non-zero susceptibility contributions: the normal polarization driven by normal fields, the normal polarization driven by tangential fields, and the tangential polarization driven by mixed fields. This allows us to write the nonlinear source vector as:

$$P_{surf}^{2\omega} = (\chi_{nnn} E_n^{\omega} E_n^{\omega} + \chi_{ntt} E_t^{\omega} E_t^{\omega})\hat{n} + (\chi_{tnt} E_n^{\omega} E_t^{\omega})\hat{t} \qquad (S5)$$

The terms χ_{nnn}, χ_{ntt}, and χ_{tnt} denote the local nonlinear susceptibility components. The nonlinear susceptibility values of gold were obtained from the experimental data [4]. To determine the far-field emission, we calculate the interaction of this source with the detector-side field. The scalar integrand is obtained by taking the dot product of this polarization vector with the reciprocal electric field vector $E^{2\omega} = E_n^{2\omega}\hat{n} + E_t^{2\omega}\hat{t}$. Expanding the term yields the final expression for the radiated field:

$$E_{SH}^{2\omega} \propto \iint (\chi_{nnn} E_n^{\omega} E_n^{\omega} E_n^{2\omega} + \chi_{ntt} E_t^{\omega} E_t^{\omega} E_n^{2\omega} + \chi_{ttn} E_n^{\omega} E_t^{\omega} E_t^{2\omega}) d^2 r \qquad (S6)$$

Supplemental Note 3: Simulation and Optimization

We used Tidy3D's FDTD solver to numerically solve Maxwell's Equations for a system comprising gold (Au) meta-atoms in air (n =1) situated on a silicon dioxide (SiO_2) substrate. A 2 nm titanium (Ti) adhesion layer is included between the Au meta-atoms and the substrate to ensure agreement with experimentally fabricated samples. The wavelength dependent complex refractive indices of Au, Ti, and SiO_2 are taken from Tidy3D's built-in material library.

The meta-atoms are arranged in a square lattice with a periodicity of 500 nm, modeled using periodic boundary conditions in x and y to imitate an infinite extended array in the x-y plane. Perfectly matched layers (PML) are implemented above and below the meta-atom plane along the z axis and are positioned at distances larger than twice the wavelength away from the structure to ensure effective absorption of the outgoing scattered light (**Fig. S2a**).

The system is excited by a plane wave incident normal to the surface, launched from the air side. The incident electric field is polarized along either the horizontal (x-pol) or vertical (y-pol) directions, unless otherwise specified.

The computational domain is discretized using Tidy3D's built in adaptive meshing algorithm with locally refined mesh elements in and around the metallic nanostructures to accurately resolve subwavelength geometric features and strong near field gradients (**Fig. S2b**). Frequency domain field monitors are placed on all surfaces of the Au meta-atoms to extract the complex electric and magnetic fields for analysis.

The nonlinear optical response is evaluated using a two-step simulation procedure. First, the structure is excited by a plane wave at the pump wavelength, and the resulting electromagnetic fields are used to calculate nonlinear polarization at each point on the surface of the meta-atom. In

a second simulation, the structure is excited by a plane wave at the emission (second-harmonic) wavelength. The overlap integral between the nonlinear polarization and the electromagnetic modes at the emission frequency is then evaluated to compute the generated second-harmonic intensity, following Equation S6.

A series of preliminary simulations were performed to determine the ideal effective length (L_{eff}) shown in **Fig. S3a** to position the plasmonic resonance near our target wavelength of 830 nm. The relationship between L_{eff} and the resonant wavelength is shown in **Fig. S3b** demonstrating a roughly linear relationship between the two variables. Once L_{eff} was determined, the asymmetry of the U ring was varied to determine the asymmetry ratio ($\frac{L_{arm}}{L_{eff}}$) that maximized SH intensity. Crucially, the total surface area of the meta-atom was held constant during this sweep to ensure that any changes in intensity resulted from mode matching rather than variations in material volume. The resulting SH intensity as a function of the asymmetry ratio is shown in **Fig. S3c**, revealing a maximum at a ratio between 0.13 and 0.15. In our particular case, we picked an asymmetry ratio of 0.147 for the final design taking fabrication errors into account.

To elucidate the physical origin of this enhancement, we analyzed the spatial distribution of the nonlinear source overlap on the meta-atom surface. **Fig. S3d** displays the surface maps of the scalar product $|E_{\omega}^2 \cdot E_{2\omega}|$ for asymmetry ratios of 0.01, 0.15, and 0.30. While the final far-field emission depends on the complex vector summation of these contributions (where phase variation can lead to destructive interference), these magnitude maps are useful for visualizing the spatial localization of the nonlinear interaction. The surface maps reveal that for all asymmetry ratios, the nonlinear interaction originates primarily from the resonator edges. However, the scalar product magnitude is maximized for an asymmetry ratio of around 0.15. This strong local excitation,

combined with the observed peak in radiated power in **Fig. S3c**, confirms that the fields interfere constructively to yield the maximum second-harmonic emission.

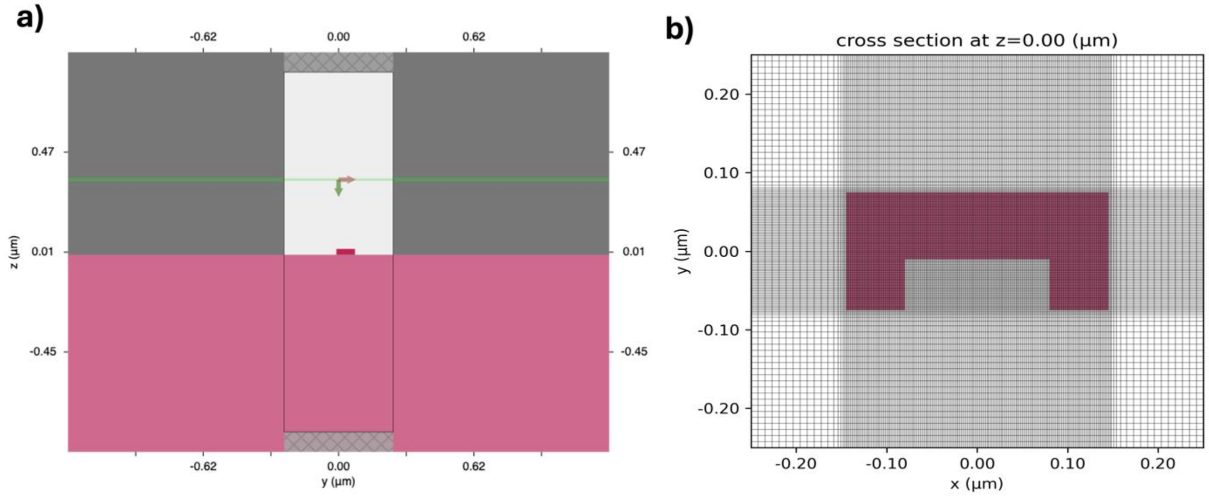

Figure S2. Simulation setup. (a) Side view of the simulation domain illustrating the structure, boundary conditions and the plane wave source. (b) Top-down view of the meshed structure.

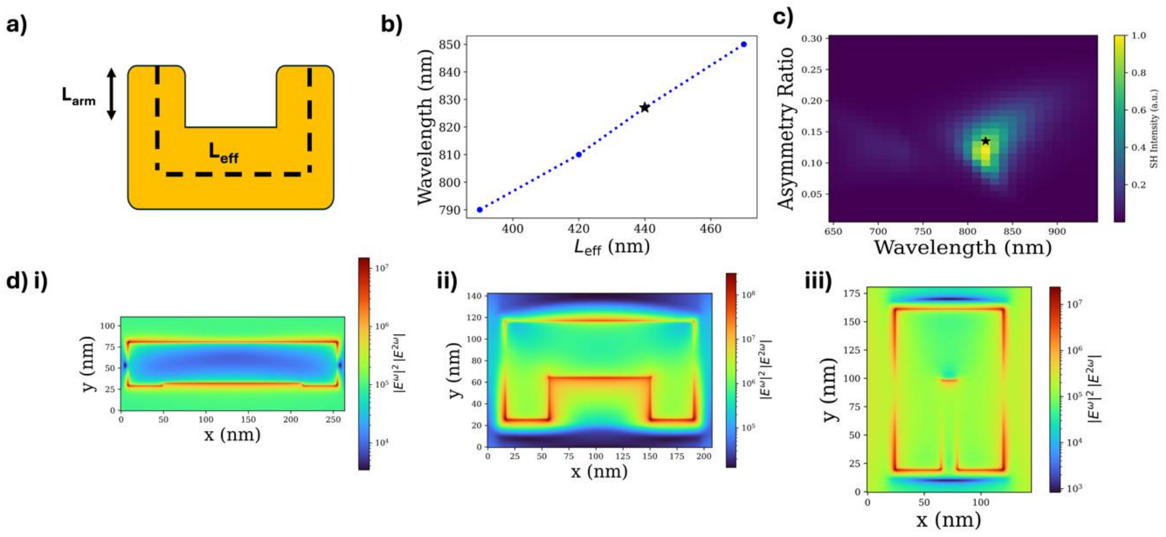

Figure S3. **Design Optimization.** (a) Schematic showing the main parameters used for optimization. (b) Plot showing the linear relationship between L_{eff} and the resonant wavelength. The black star indicates the L_{eff} that we used for our design. (c) Colormap showing the relationship between the asymmetry ratio, wavelength and the normalized SH intensity. The black star indicates the asymmetry ratio that we used for our design. (d) Surface colormap of the scalar product $|E_{\omega}^{2} \cdot E_{2\omega}|$ for different asymmetry ratios: (i) 0.01, (ii) 0.15, (iii) 0.30.

Supplementary Note 4: Sample Preparation

The samples were prepared using a standard electron beam lithography (EBL) and metal lift-off process in the Integrated Nanosystems Center at the University of Rochester. The metasurface array was fabricated on a quartz substrate. First, a 60-nm-thick layer of polymethyl methacrylate (PMMA-A2) was spin-coated on the substrate, followed by the deposition of a discharge H2O conductive layer for Electron Beam Lithography (EBL). Next, the nanometer-scale metamaterial pattern was exposed onto the resist using high-resolution EBL (Elionix ELS-S50EX). The pattern was then rinsed with deionized water, and the development was carried out using a MIBK: IPA 1:3 solution. Following the development process, the KLJ PVD-75 electron beam evaporation system was used to deposit titanium and gold thin films. The gold and titanium layers had thicknesses of 35 nm and 2 nm, respectively, with the titanium layer serving to improve the adhesion between the gold layer and the quartz substrate. Finally, acetone was used to gently lift off the photoresist layer.

Supplementary Note 5: Experimental Setup for Ultrafast Pulse Characterization

To demonstrate the practical utility of the designed metasurface as a nonlinear medium, we integrated it into a Michelson-type interferometer for ultrashort pulse characterization (**Fig. S4**). To record a fringe-resolved interferometric autocorrelation (IAC) of a Ti: sapphire oscillator, the femtosecond beam first passed through a half-wave plate and polarizer to precisely set the input polarization for optimal SHG. This polarized beam was then directed into a Michelson-type interferometer, creating two collinear replicas with a variable time delay (introduced by a mirror on a translation stage). The recombined beam pair was focused onto the metasurface by a lens. The resulting SH signal was first collected and collimated, then spectrally filtered to reject the fundamental, and finally recorded by a PMT as a function of the delay.

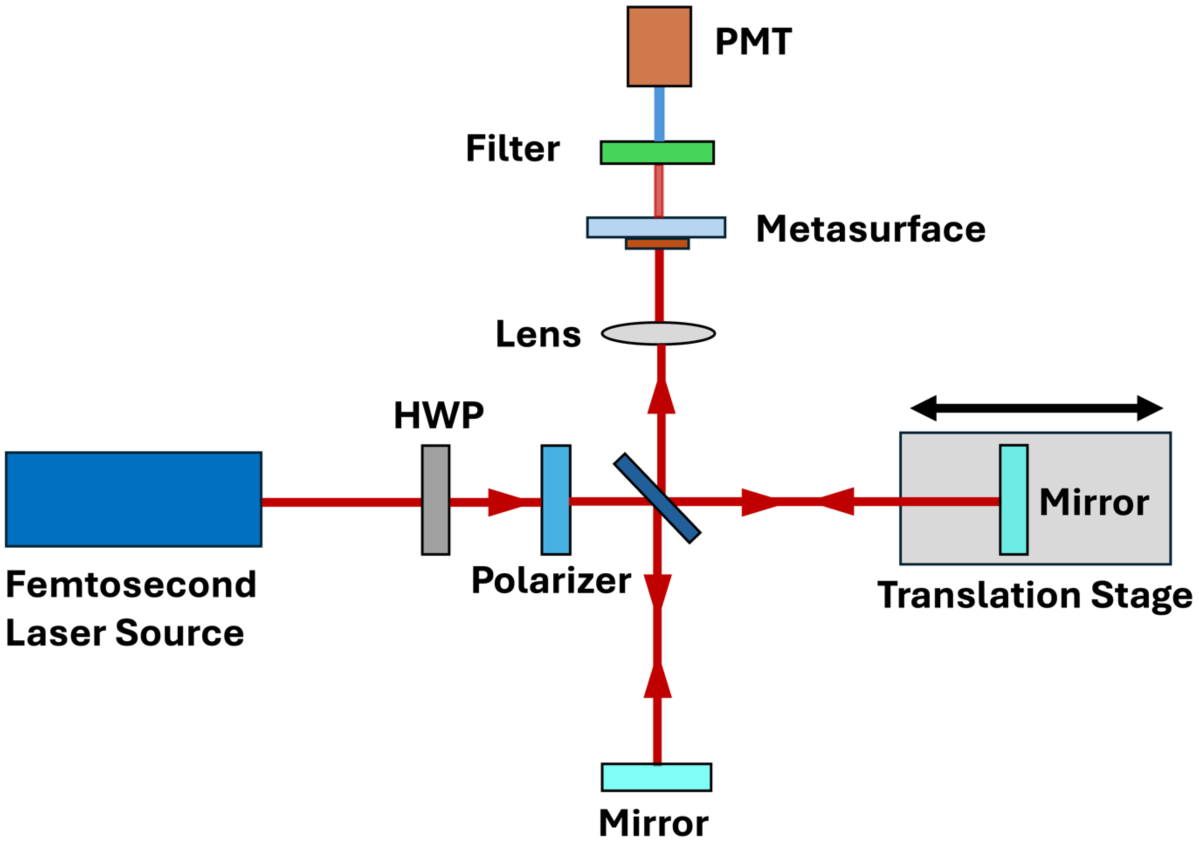

Figure S4. Schematic of the experimental setup for measuring the fringe-resolved interferometric autocorrelation (IAC) trace.

Supplementary Note 6: Derivation of the Peak-to-Background Ratio for Interferometric Autocorrelation.

The collinear geometry present in the interferometric autocorrelation provides a robust way to validate the measurement of the second-harmonic autocorrelation signal. The electric field of the second-harmonic signal, $E_{2\omega}(t,\tau)$, generated by two collinear replica pulses separated by temporal delay τ, is proportional to the square of the sum of the incident fields.

$$E_{2\omega}(t,\tau) \propto [E_{\omega}(t) + E_{\omega}(t-\tau)]^2 \qquad (S1)$$

The intensity signal recorded by the slow detector, $I(\tau)$, corresponds to the time integrated intensity of the second harmonic field.

$$I(\tau) \propto \int_{-\infty}^{\infty} |[E_{\omega}(t) + E_{\omega}(t-\tau)]^2|^2 dt \qquad (S2)$$

To determine the peak-to-background contrast ratio, we evaluate this integral at two limiting cases:

1. At Zero Delay ($\tau = 0$): When the pulses overlap perfectly in time and phase, the fields add constructively. The total field amplitude doubles, and the nonlinearity squares this sum:

$$E_{2\omega}(t,0) \propto [2E_{\omega}(t)]^2 = 4E_{\omega}(t) \qquad (S3)$$

The detected peak intensity is therefore:

$$I_{peak} \propto 16 \int_{-\infty}^{\infty} |E_{\omega}(t)|^4 dt \qquad (S4)$$

2. At Infinite Delay ($\tau \rightarrow \infty$): When the delay is large ($\tau \gg \tau_{\text{pulse}}$), the pulses are temporally separated and do not overlap. The integral separates into the sum of the individual second-harmonic intensities generated by each pulse independently.

$$I_{bg} \propto \int_{-\infty}^{\infty} |E_\omega(t)|^4 dt + \int_{-\infty}^{\infty} |E_\omega(t-\tau)|^4 dt = 2\int_{-\infty}^{\infty} |E_\omega(t)|^4 dt \qquad (S5)$$

Thus, the theoretical ratio between the peak and background intensities is 8:1. Observation of this ratio in the experimental data is the indicator of precise collinear alignment of the setup and validates the fidelity of the interferometric measurement [5].

Supplementary Note 7: PCGP Retrieval Algorithm

The pulse retrieval is performed using the Principal Components Generalized Projections (PCGP) algorithm[6], as outlined in the flowchart (**Fig. S5**). The algorithm is initialized with a random guess for the signal pulse, $S(t)$, and the gate pulse, $G(t)$. In each iteration, the outer product matrix $O(t_1, t_2) = S(t_1) \otimes G(t_2)$ is constructed and subsequently converted into the time-delay signal domain, $E(t, \tau)$, via a row-shifting coordinate transformation. This signal is Fourier transformed into the frequency domain, $E(\omega, \tau)$, where the experimental constraint is applied by replacing the magnitude of the calculated signal with the square root of the measured FROG trace, while preserving the calculated phase. After applying the inverse Fourier transform to the modified electric field, $E'(\omega, \tau)$, the signal is shifted back to the outer product domain via an inverse coordinate transformation. The resulting matrix, $O_{new}(t_1, t_2)$, is typically full-rank tensor and does not correspond to a single physical pulse pair. To enforce the physical constraint, a rank-1 approximation is performed using the Singular Value Decomposition (SVD) method. This step extracts the principal singular vectors, $S_{new}(t)$ and $G_{new}(t)$, which minimizes the deviation from the modified matrix and serves as the updated inputs. The cycle repeats until the RMS error between the retrieved and measured traces converges to a minimum.

To ensure reliable pulse reconstruction, we utilized the standard Principal Component Generalized Projections (PCGP) algorithm via the open source froglib package [7]. We rigorously validated this implementation by performing a blind retrieval test on a synthetic pulse with temporal and spectral characteristics similar to our experimental signal. We generated a theoretical FROG trace from a known reference pulse, added Gaussian noise to simulate experimental conditions, and successfully retrieved the original amplitude and phase with high accuracy, achieving a final FROG error of 9×10^{-5} (**Fig. S6**). This benchmark confirms the algorithm's

reliability for our specific pulse regime. For the experimental data, we monitored the error evolution (**Fig. S7**) and achieved the FROG error of 1×10^{-5}, observing a monotonic convergence to a stable minimum.

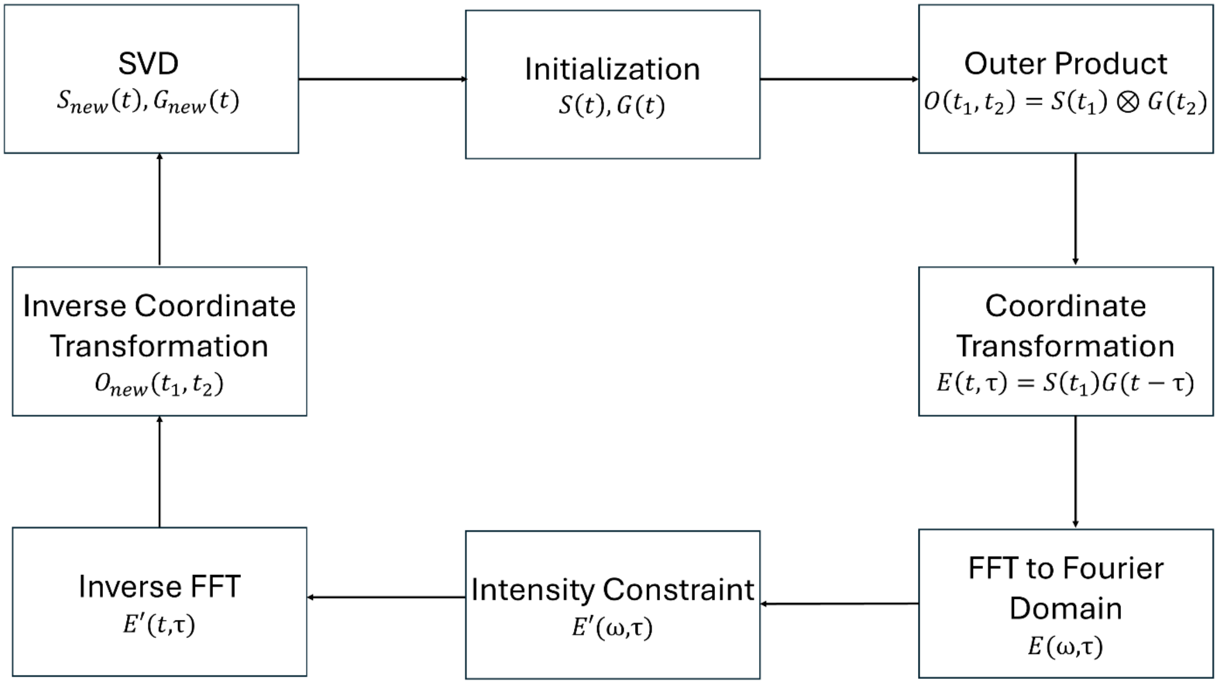

Figure S5. Flowchart of the Principal Components Generalized Projections (PCGP) algorithm used for pulse retrieval.

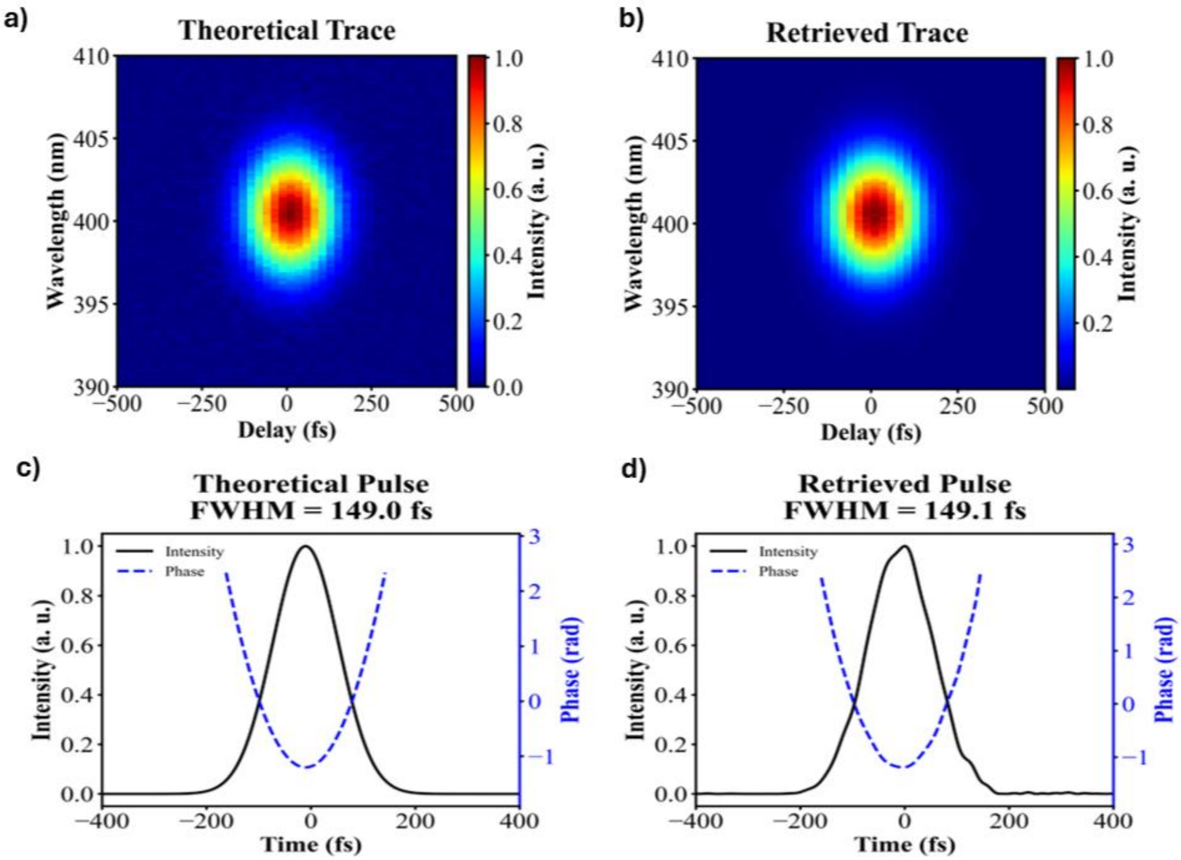

Figure S6. Validation of the PCGP retrieval algorithm. (a) Theoretical SHG FROG trace generated from a 150-fs chirped Gaussian pulse with added Gaussian noise. (b) Retrieved FROG trace using the Principal Component Generalized Projections (PCGP) algorithm via the *froglib* package. (c) and (d) Comparison of the theoretical (black) and retrieved (blue dashed) temporal intensity and phase profiles, showing excellent agreement.

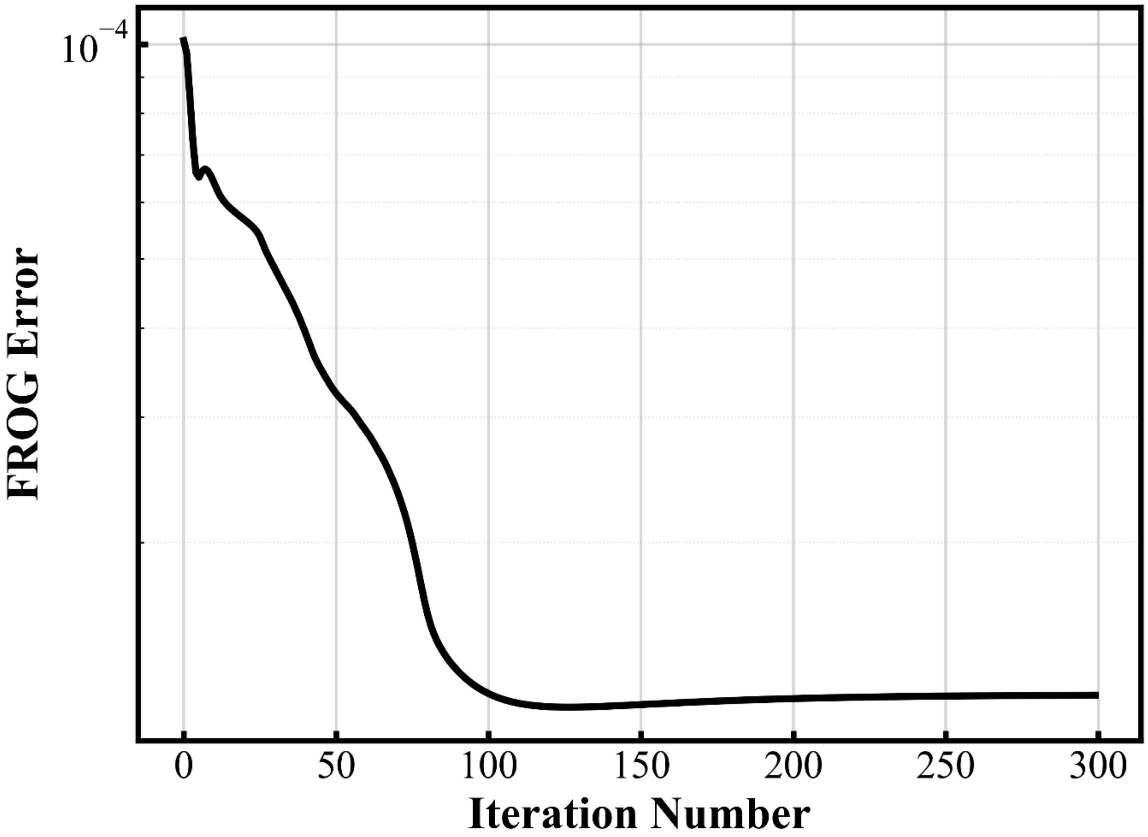

Figure S7. Convergence of the PCGP retrieval algorithm. The plot displays the evolution of the FROG error as a function of iteration number for the retrieved pulse presented in the main text.

REFERENCES

(1) Boyd, R. W. *Nonlinear optics*; Elsevier, 2003.

(2) Krasnok, A.; Tymchenko, M.; Alù, A. Nonlinear metasurfaces: a paradigm shift in nonlinear optics. *Materials Today* **2018**, *21* (1), 8–21. DOI: https://doi.org/10.1016/j.mattod.2017.06.007.

(3) O'Brien, K.; Suchowski, H.; Rho, J.; Salandrino, A.; Kante, B.; Yin, X.; Zhang, X. Predicting nonlinear properties of metamaterials from the linear response. *Nature Materials* **2015**, *14* (4), 379–383. DOI: 10.1038/nmat4214.

(4) Wang, F. X.; Rodríguez, F. J.; Albers, W. M.; Ahorinta, R.; Sipe, J. E.; Kauranen, M. Surface and bulk contributions to the second-order nonlinear optical response of a gold film. *Physical Review B* **2009**, *80* (23), 233402. DOI: 10.1103/PhysRevB.80.233402.

(5) Diels, J.-C.; Rudolph, W. Ultrashort Laser Pulse Phenomena Fundamentals, Techniques, and Applications an a Femtosecond Time Scale Second Edition. **1996**.

(6) Kane, D. J. Principal components generalized projections: a review [Invited]. *J. Opt. Soc. Am. B* **2008**, *25* (6), A120–A132. DOI: 10.1364/JOSAB.25.00A120.

(7) Mahnke, C. *froglib*. GitHub, 2018. https://github.com/xmhk/froglib (accessed).
